# Identification of the co-differentially expressed hub genes involved in the endogenous protective mechanism against ventilator-induced diaphragm dysfunction

**DOI:** 10.1186/s13395-022-00304-w

**Published:** 2022-09-09

**Authors:** Dong Zhang, Wenyan Hao, Qi Niu, Dongdong Xu, Xuejiao Duan

**Affiliations:** 1 Department of Critical Care Medicine, Heping Hospital Affiliated to Changzhi Medical College, 110 South Yan’an Road, Luzhou District, Changzhi, 046012 China; 2 Department of Biomedical Engineering, Changzhi Medical College, Changzhi, 046012 China

**Keywords:** Diaphragm dysfunction, Mechanical ventilation, Endogenous protective mechanism, Weighted gene coexpression network analysis, Co-differentially expressed hub genes

## Abstract

**Background:**

In intensive care units (ICU), mechanical ventilation (MV) is commonly applied to save patients’ lives. However, ventilator-induced diaphragm dysfunction (VIDD) can complicate treatment by hindering weaning in critically ill patients and worsening outcomes. The goal of this study was to identify potential genes involved in the endogenous protective mechanism against VIDD.

**Methods:**

Twelve adult male rabbits were assigned to either an MV group or a control group under the same anesthetic conditions. Immunostaining and quantitative morphometry were used to assess diaphragm atrophy, while RNA-seq was used to investigate molecular differences between the groups. Additionally, core module and hub genes were analyzed using WGCNA, and co-differentially expressed hub genes were subsequently discovered by overlapping the differentially expressed genes (DEGs) with the hub genes from WGCNA. The identified genes were validated by western blotting (WB) and quantitative real-time polymerase chain reaction (qRT–PCR).

**Results:**

After a VIDD model was successfully built, 1276 DEGs were found between the MV and control groups. The turquoise and yellow modules were identified as the core modules, and *Trim63*, *Fbxo32*, *Uchl1*, *Tmprss13*, and *Cst3* were identified as the five co-differentially expressed hub genes. After the two atrophy-related genes (*Trim63* and *Fbxo32*) were excluded, the levels of the remaining three genes/proteins (*Uchl1*/UCHL1, *Tmprss13*/TMPRSS13, and *Cst3*/CST3) were found to be significantly elevated in the MV group (*P* < 0.05), suggesting the existence of a potential antiproteasomal, antiapoptotic, and antiautophagic mechanism against diaphragm dysfunction.

**Conclusion:**

The current research helps to reveal a potentially important endogenous protective mechanism that could serve as a novel therapeutic target against VIDD.

## Background

During the coronavirus disease 2019 (COVID-19) pandemic, mechanical ventilation (MV) has been of critical importance to save patients suffering from severe hypoxemic respiratory failure [1]. However, studies involving rat models have shown that an MV period as short as 12 h can decrease both the size of the diaphragm fibers and the generation of a specific force in the diaphragm in a condition called ventilator-induced diaphragmatic dysfunction (VIDD) [2]. VIDD is primarily characterized by an early loss of diaphragm contractility that is later followed by muscle atrophy [3, 4]. In some patients, disuse-induced atrophy can even cause an average reduction in diaphragmatic thickness of 6% per day [5]. Furthermore, the development of VIDD is associated with numerous complications, including ventilator-associated pneumonia, increased hospital mortality, and poor clinical prognosis in critically ill patients, making weaning from the ventilator increasingly difficult [6, 7].

Given that VIDD results from impaired protein synthesis and increased proteolysis, understanding these processes can be vital for protecting the diaphragm [8]. In this context, it has been reported that after 12 h of MV, protein synthesis is downregulated in the diaphragm through disruption of the insulin-like growth factor (IGF)/protein kinase B (AKT)/mammalian target of rapamycin (mTOR) signaling pathway [9]. Moreover, during MV, increased reactive oxygen species (ROS) levels in the mitochondria can activate all the major proteolytic systems (the calpain system, apoptosis, the ubiquitin–proteasome system [UPS], and autophagy) in the diaphragm, with this process significantly contributing to proteolysis [10, 11]. As a result, diaphragmatic protein homeostasis can be disrupted, leading to muscle atrophy. In cellular processes, protein quality control and homeostasis are ensured by the UPS. However, studies on the diaphragms of both rats and humans have shown that MV causes muscle-specific E3 ligases (atrogin1/muscle atrophy F-box and MuRF1/muscle ring finger-1) to be increasingly expressed [12], which has also been reported to cause 20S proteasome activity to increase [13]. Altogether, these findings indicate that MV induces the UPS to be increasingly activated in the diaphragm. Another catabolic process that could be involved in VIDD is autophagy, in which cytosolic proteins and organelles are degraded by lysosomes. MV has been shown to increase autophagy in the diaphragms of both humans and animals, and due to the clearance of catalase and peroxisomes, this eventually impairs the antioxidant capability of cells. Another study found that a number of major autophagy genes (*beclin1*, *Atg7*, and *Atg5*) are upregulated by MV and that the expression of the autophagosome marker microtubule-associated protein light chain 3-II (LC3B) is concomitantly increased [14, 15], supporting the notion that autophagy is indeed essential for VIDD. Additional evidence suggests that MV can even cause apoptosis in the rat diaphragm by activating caspase-3 [16], a cysteine protease that further activates nucleases to damage double-stranded DNA and cause the loss of myonuclei. The apoptotic process is also mediated by the Bcl2-interacting mediator of cell death (Bim) in the case of MV in humans [17]. These results further demonstrate the involvement of apoptosis in the progression of VIDD. Calpain activation is another factor that is directly involved in VIDD via the promotion of rapid proteolysis. For instance, in the sarcoplasmic reticulum (SR), modification of a ryanodine receptor/Ca^2+^ release channel due to high levels of ROS from mitochondria causes the release of Ca^2+^ from the SR and the activation of calpains [18]. Additionally, as specialized proteases that assist in cell communication by cleaving target proteins into physiologically active fragments, calpains may act within the cytoplasm of diaphragm fibers to cleave PKC-δ and DRP1 into fragments that subsequently take part in MV-induced mitochondrial dysfunction [19]. Overall, the above findings support the idea that MV-associated fiber atrophy in diaphragms results from increased proteolysis due to high levels of active calpains, elevated UPS activity, and increased apoptosis and autophagy.

While a number of studies have considered the processes behind VIDD, only a few have investigated the endogenous protective mechanism against it. In this context, a study on newborn lamb diaphragms during MV showed that increased expression of the histone deacetylase Sirtuin1 (*Sirt1*) acts as a protective factor by downregulating atrophy-inducing genes such as *Atrogin1* and *Murf1* [20]. Similarly, increased levels of heat shock proteins (HSPs) due to stress are vital for protection against limb muscle loss from immobilization. Generally, diaphragm HSPs are responsible for maintaining muscle integrity while promoting muscle regeneration and recovery, and HSP72, in particular, can protect against VIDD by inhibiting the activity of transcription factors such as nuclear factor kappa B (NF-κB) and forkhead box O (FoxO) to reduce oxidative stress in cells [21, 22]. In VIDD, the renin–angiotensin system (RAS) causes oxidative stress and induces atrophy via its main peptide angiotensin II. However, RAS also possesses a counterregulatory axis in which the peptide angiotensin (1–7) reduces oxidative stress and proteolysis, thereby protecting against the onset of VIDD [23, 24]. Although the results of prior studies clearly show that SIRT1, HSP72, and angiotensin (1–7) are essential for maintaining diaphragm function in VIDD, the intrinsic antiautophagic, antiapoptotic, and antiproteasomal mechanisms for protecting the diaphragm during MV remain unclear. Therefore, it is important to identify hallmark genes that could be involved in the above protective mechanisms.

In 2005, weighted gene coexpression network analysis (WGCNA) was proposed as a method for identifying potential biomarkers based on interconnections among a subset of genes and their associations with phenotypes [25]. Through this approach, one group of researchers identified a coexpression network of three mRNAs involved in muscle atrophy (*Myog*, *Trim63*, and *Fbxo32*) and several newly discovered long noncoding RNAs (NONRATT026958.2, NONRATT026957.2, and NONRATT008228.2, among others) that are essential for VIDD [26]. In this study, in addition to high-throughput RNA sequencing (RNA-seq) to identify differentially expressed genes (DEGs) between the control (CON) and MV-treated groups, WGCNA was undertaken to explore the core modules in the diaphragm after MV. By overlapping the DEGs and the hub genes from the core module, a set of common genes, labeled as co-differentially expressed hub genes, were identified. Although the diaphragm transcriptome has been profiled in MV models and the activities of specific miRNAs have been studied [27], little is known about potential internal protective mechanisms against VIDD. Therefore, based on high-throughput RNA-seq and WGCNA, the current study aimed to uncover co-differentially expressed hub genes in the diaphragm that could be involved in an endogenous mechanism during MV.

## Methods

### Animals

Adult male rabbits weighing 2.2 to 2.5 kg were obtained from Shanxi Medical University (Taiyuan, China) and housed in an environment with a 12-h light/dark cycle and unrestricted access to food and water. The animal experiments were approved by the Ethics Committee of Changzhi Medical College and conducted according to the Guide for the Care and Use of Laboratory Animals.

### Model of ventilator-induced diaphragm dysfunction (VIDD)

**Fig. 1:**
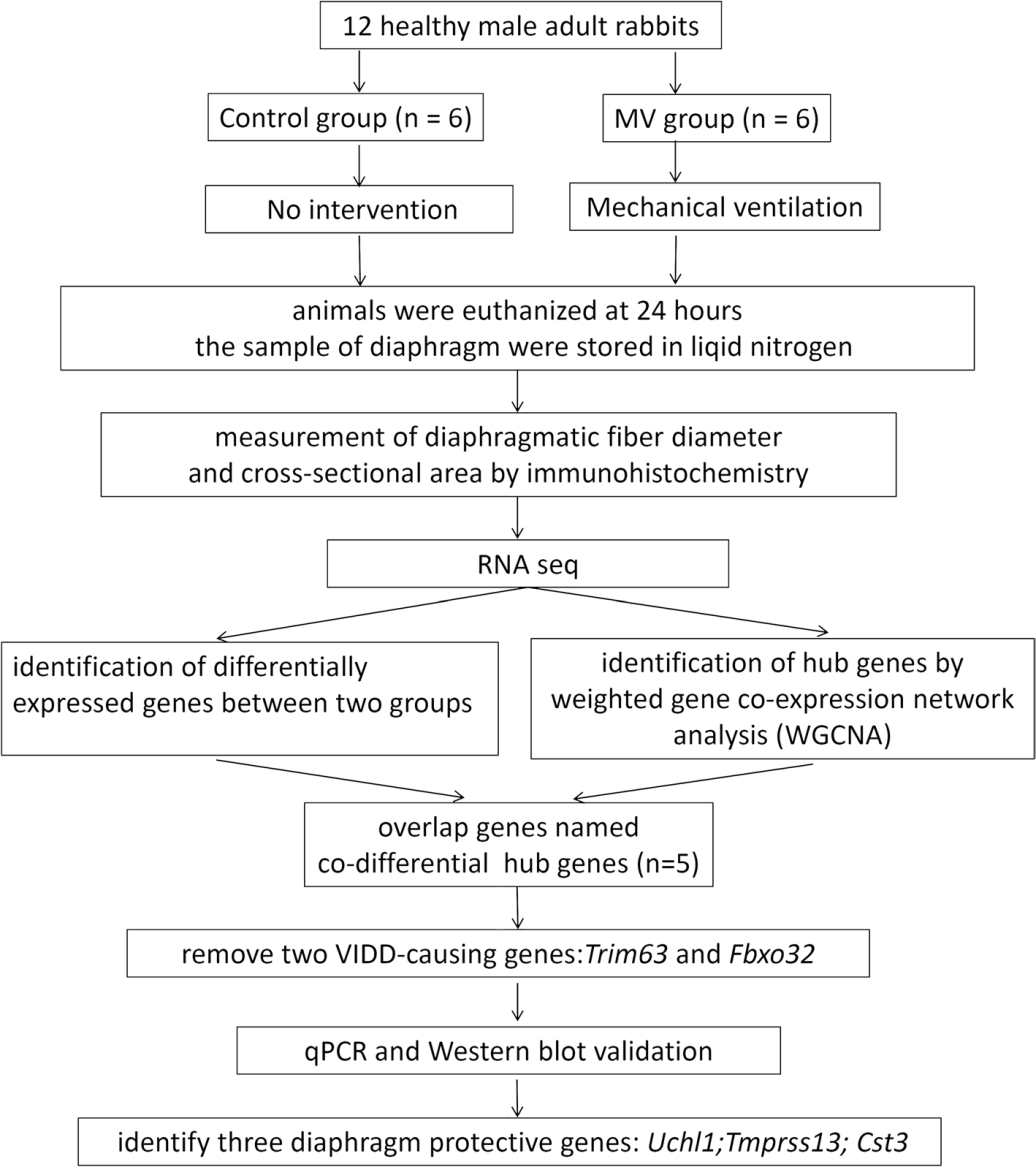
Diagram of the strategies for determining the co-differentially expressed hub genes

Twelve adult male rabbits were randomly divided into two groups. In the control group (*n* = 6), the animals received an anesthetic but were not kept under ventilation, while in the MV group (*n* = 6), the animals received MV for 24 h (Fig. 1). Sodium pentobarbital (40 mg/kg) was then administered intraperitoneally to anesthetize the rabbits, and the drug was allowed to continuously infuse (10 mg/kg/h) using an electric pump. Next, tracheostomy was performed to connect the animals to small ventilators (Inspira ASVV 55-7058; Harvard Apparatus, Cambridge, MA, USA) under a respiratory rate, tidal volume (Vt), and positive end-expiratory pressure of 40 cycles per minute, 6 mL/kg, and 2 cmH_2_O, respectively. In addition, the jugular vein and carotid artery were cannulated to measure blood pressure and administer anesthetics continuously. Throughout the ventilation process, the animals’ body temperature was maintained at 37 °C using a homeothermic blanket system, and the levels of arterial blood gasses were monitored (ABL80 FLEX; Radiometer Medical, Carlsbad, CA, USA) to ensure that the PaCO_2_ was between 35 and 45 mmHg and the PaO_2_ was between 80 and 100 mmHg. Upon completion of the MV period, the animals from both groups were sacrificed. Biopsy samples were then taken quickly from the middle portion of the right costal diaphragm. One strip was retained for histological assessment, while the rest was quickly frozen in liquid nitrogen and stored at −80 °C until further analysis.

### Histological measures

After muscle samples were fixed in 4% paraformaldehyde, they were embedded in paraffin and cut into sections. The sections were subsequently dewaxed and rehydrated prior to staining with hematoxylin–eosin (HE). Immunostaining was then performed by blocking the sections for 1 h using 2% bovine serum albumin (BSA), incubating them overnight at 4 °C with mouse anti-fast myosin skeletal heavy chain (ab51263, Abcam, Cambridge, UK) as the primary antibody, washing them three times with PBS, and subsequently incubating them for 1 h at room temperature with goat anti-rabbit IgG (ab205719, Abcam) as the secondary antibody. Images of the stained samples were acquired using a BX41 microscope (Olympus, Tokyo, Japan), and an investigator blinded to the genotype status of the samples used Image-Pro Plus version 6.0 software (Media Cybernetics, Bethesda, MD, USA) to measure the diameter and cross-sectional area (CSA) of the muscle fibers.

### RNA sequencing (RNA-seq) and bioinformatic analysis

High-throughput RNA-seq was applied to investigate changes in mRNA expression. TRIzol reagent (15596026; Ambion, Austin, TX, USA) was first used to extract the total RNA from diaphragm tissues before RNA integrity was determined on an Agilent 2100 Bioanalyzer (Thermo Fisher Scientific, Waltham, MA, USA). A value ≥ 7 for the RNA integrity number (RIN) was selected as the criterion for inclusion in subsequent experiments. After library preparation and sequencing on an MGISEQ-2000 sequencing platform, data analysis was performed on the Dr. Tom platform (http://report.bgi.com; Huada Gene Science and Technology Service Co., Ltd., Shenzhen, China). DEGs between the MV and control groups were subsequently identified with a *Q* value < 0.05 and a |log_2_FC| > 1 as the thresholds. In addition, the distribution of DEGs was illustrated with volcano plots, and the top 40 DEGs were illustrated with a heatmap. The hub genes within the core modules related to MV were explored using the WGCNA package (1.70–3) in R (version 4.0.5). The module eigengene (ME) is the main component representing coexpressed genes in a module. Since the ME can reflect the features of all genes in a module, correlational analysis between the binary status of the treatment (the features of the control and MV groups ) and the ME was performed using Pearson’s correlation tests. Modules with a *P* value of < 0.05 and the highest correlation coefficient were subsequently selected. Module membership (MM) was defined as a correlation between an individual gene and the ME. In addition, gene significance (GS) represents the correlation between a gene and group features. The hub genes within the modules were identified as those with an MM>0.8 and a GS>0.8. A Venn diagram tool (https://bioinfogp.cnb.csic.es/tools/venny/) was applied to perform intersection analysis between the DEGs and the hub genes in the core modules. A coexpression network of the co-differentially expressed hub genes was built and then visualized using Cytoscape 3.8.2 (www.cytoscape.org).

### Quantitative real-time polymerase chain reaction (qRT–PCR)

The results obtained from the RNA-seq experiment were validated by qRT–PCR for the three genes *Uchl1*, *Tmprss13*, and *Cst3*. Total RNA extraction was performed as described above, followed by cDNA synthesis using PrimeScript II Reverse Transcriptase (2690A, Takara Bio USA Inc., San Jose, CA, USA). qRT–PCR was subsequently carried out on a CFX-Connect real-time PCR detection system (Bio-Rad, Hercules, CA, USA) by adding 1 μL of cDNA to 20 μL of the reaction mixture from the SYBR FAST qPCR Master Mix kit (KM4101, Kapa Biosystems, Woburn, MA, USA). In this experiment, the expression of target genes was normalized using β-actin as an internal control, while relative quantification was performed with the comparative cycle threshold method. The results are expressed as the fold changes with respect to the control values. The primer sequences (5’-3’) were as follows: *Uchl1*, forward: GTCCCCTGAAGACAGAGCAAG, reverse: GCATTCGCCCATCAAG; *Tmprss13*, forward: CATCATCAACGGCAACTACAC, reverse: CGTCTGTCTCCTTGGTCTTG; and *Cst3*, forward: GGCAGATCGTAAGTGGCG, reverse: GTCGTGGAAAGGACAGTTGG.

### Western blot analysis

Western blotting was carried out as previously described [28]. Briefly, proteins were extracted from costal diaphragm tissues using RIPA lysis buffer (R0010, Solarbio Science & Technology Co., Ltd., Beijing, China) before being quantified using a bicinchoninic acid (BCA) protein assay (PC0020, Solarbio) according to the manufacturer’s instructions. The proteins were then separated by SDS–PAGE and transferred to polyvinylidene difluoride (PVDF) membranes, which were incubated overnight at 4 °C with one of the following primary antibodies: anti-TMPRSS13 (JL-T0294, Jianglai, Shanghai, China), anti-UCH-L1 (NBP2-29420, Novus Biologicals, Littleton, CO, USA), anti-CST3 (C8950, United States Biological, Salem, MA, USA) or anti-β-actin (PAB36265, Bioswamp, Wuhan, China). On the following day, the membranes were incubated with goat anti-rabbit IgG (SAB43714, Bioswamp) as the secondary antibody for 1 h at room temperature. Finally, images of the western blots were scanned and analyzed with Tanon GIS version 4.2 (Tanon Technology, Shanghai, China) to quantify the density of the visible bands, with the results normalized relative to those for β-actin.

### Statistical analysis

Statistical analysis was carried out using SPSS 25.0. All data are expressed as the mean ± standard deviation (SD). The standard two-tailed *t* test was also used to analyze differences between the means of the two groups at the 5% significance level. The PCR data were analyzed with the control pool expression set as 1.

## Results

### Establishment of the VIDD model

**Fig. 2:**
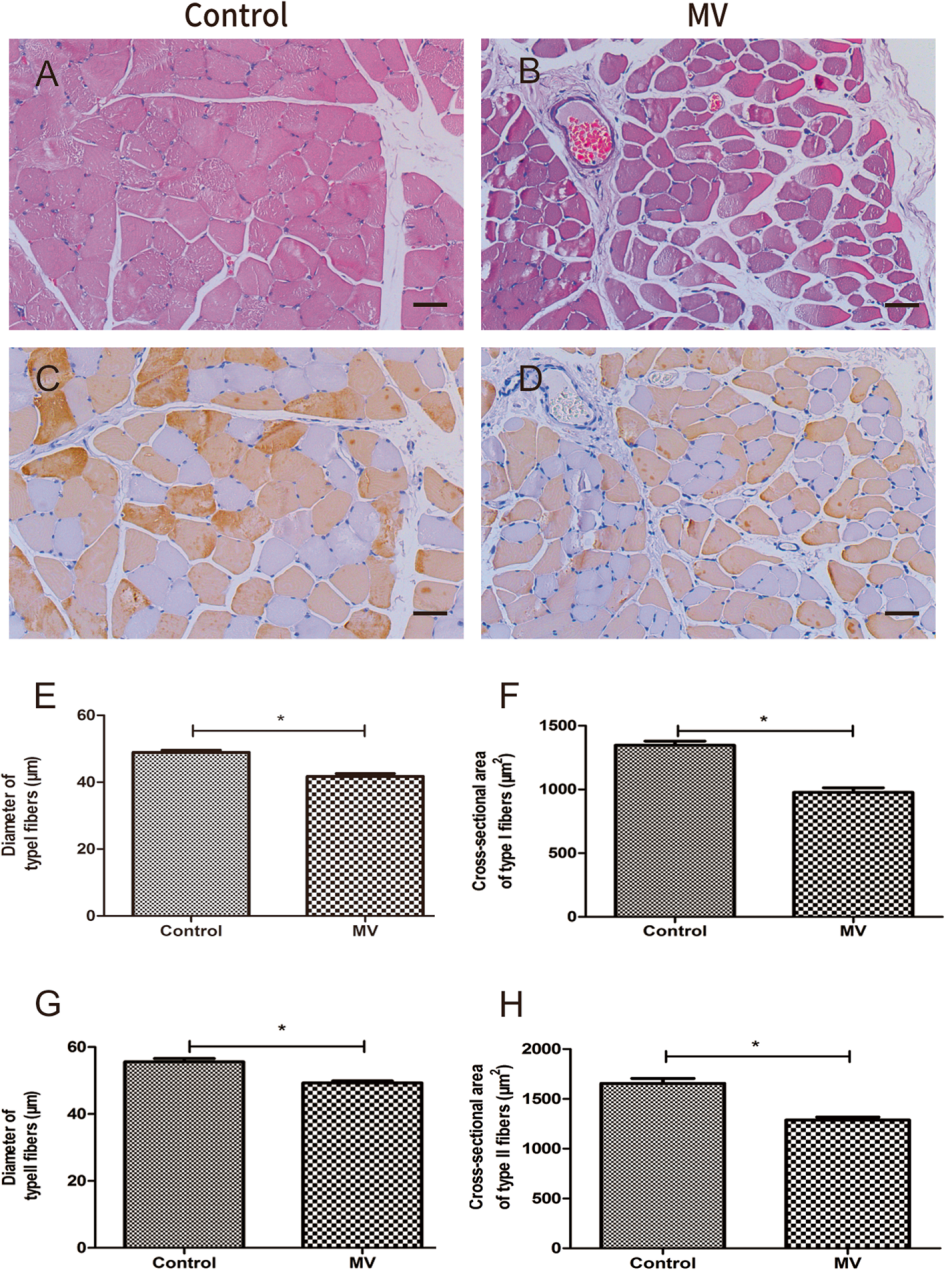
Hematoxylin–eosin (HE) staining and immunohistochemistry. **A**–**D** HE staining (top) and immunohistochemistry (bottom) of slow-twitch and fast-twitch fibers in the diaphragm of one representative rabbit for each group (control, MV). **E**–**H** Mean fiber size and cross-sectional area (CSA) of slow-twitch and fast-twitch fibers of the diaphragm muscles for the two groups. Groups: Control = control group; MV = mechanical ventilation group. *P < 0.05 with respect to the control group; scale bar = 50 μm. The error bars indicate the standard deviation (SD)

The VIDD model was successfully established. The body weights of the animals were not found to be significantly different between the two groups prior to MV. Furthermore, as shown in Table 1, the two groups showed no significant differences in terms of physiological parameters (*P* > 0.05), such as the arterial partial pressures of oxygen (PaO_2_) and carbon dioxide (PaCO_2_), systolic blood pressure (SBP), heart rate (HR), and pH, suggesting that these parameters remained relatively constant throughout the MV. However, immunohistochemistry revealed that after 24 h of MV, the diaphragm fast- and slow-twitch fibers had a significantly lower CSA and diameter in the MV group compared with the control group (Fig. 2).

**Table 1:** Comparison of physiological parameters between the two groups

	Control group (*n* = 6)		MV group (*n* = 6)	
	0 h	24 h	0 h	24 h
BP (cmH_2_O)	123 ± 7.87	118 ± 10.16	120 ± 9.67	119 ± 9.22
HR (beats/minute)	229 ± 12.14	230 ± 16.13	230 ± 12.36	231 ± 16.23
pH	7.35 ± 0.08	7.32 ± 0.07	7.35 ± 0.10	7.34 ± 0.07
PO_2_ (kPa)	119 ± 7.10	126 ± 7.82	129 ± 12.3	131 ± 10.75
PCO_2_ (kPa)	38 ± 4.15	37 ± 4.02	39 ± 2.81	37 ± 5.68

The data are expressed as the mean ± standard deviation

*BP* blood pressure, *HR* heart rate, *PaCO*_*2*_ arterial partial pressure of carbon dioxide, *PaO*_*2*_ arterial partial pressure of oxygen

### Differential gene expression analysis

**Fig 3:**
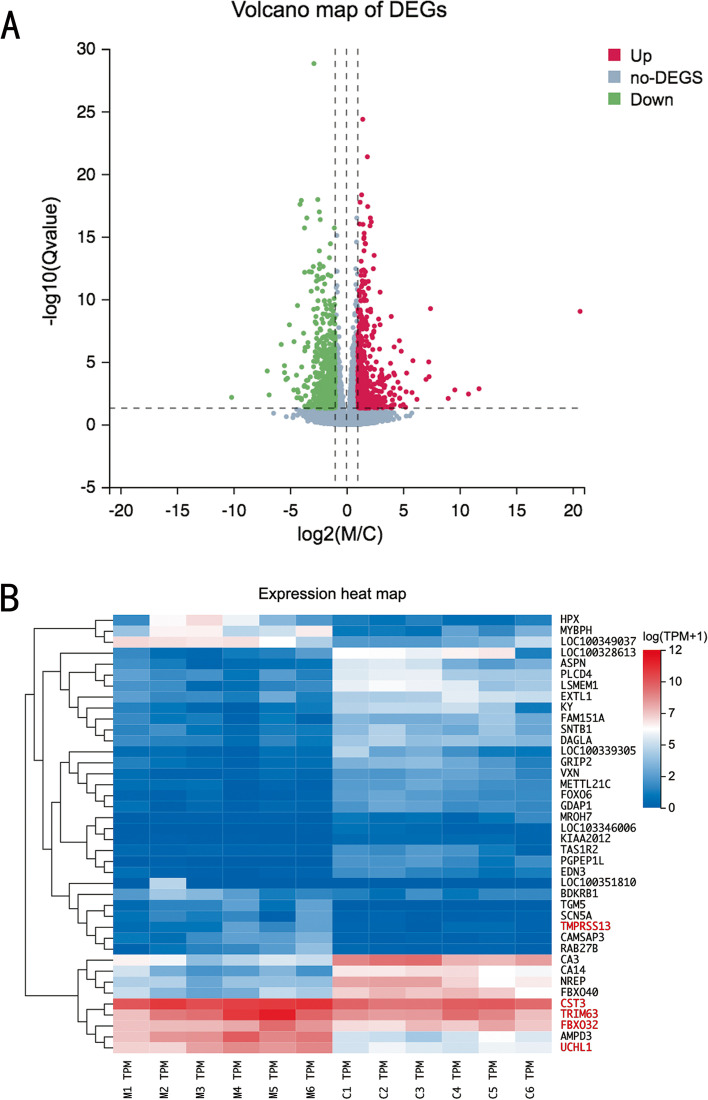
Volcano plot and heatmap of the differentially expressed genes (DEGs). **A** Volcano plot showing the 1276 DEGs between the mechanical ventilation (MV) and control groups, of which 760 were downregulated and 516 were upregulated. The cutoff criteria were a |log_2_FC|>1 and a *Q* value< 0.05. Green represents significantly downregulated genes, grey represents non-DEGs, and red represents upregulated genes. **B** Heatmap of the hierarchical clustering analysis for the top 40 DEGs between the MV and control groups. Each column represents a sample, and each row represents a single gene

RNA-seq experiments were carried out to identify genes that were differentially expressed between the two groups. In the current study, 1276 DEGs were found, of which 760 were downregulated and 516 were upregulated in the MV group compared with the control group. A volcano plot was created to display all DEGs (Fig. 3A). Furthermore, the top 40 DEGs are illustrated in a heatmap (Fig. 3B).

### WGCNA

**Fig. 4:**
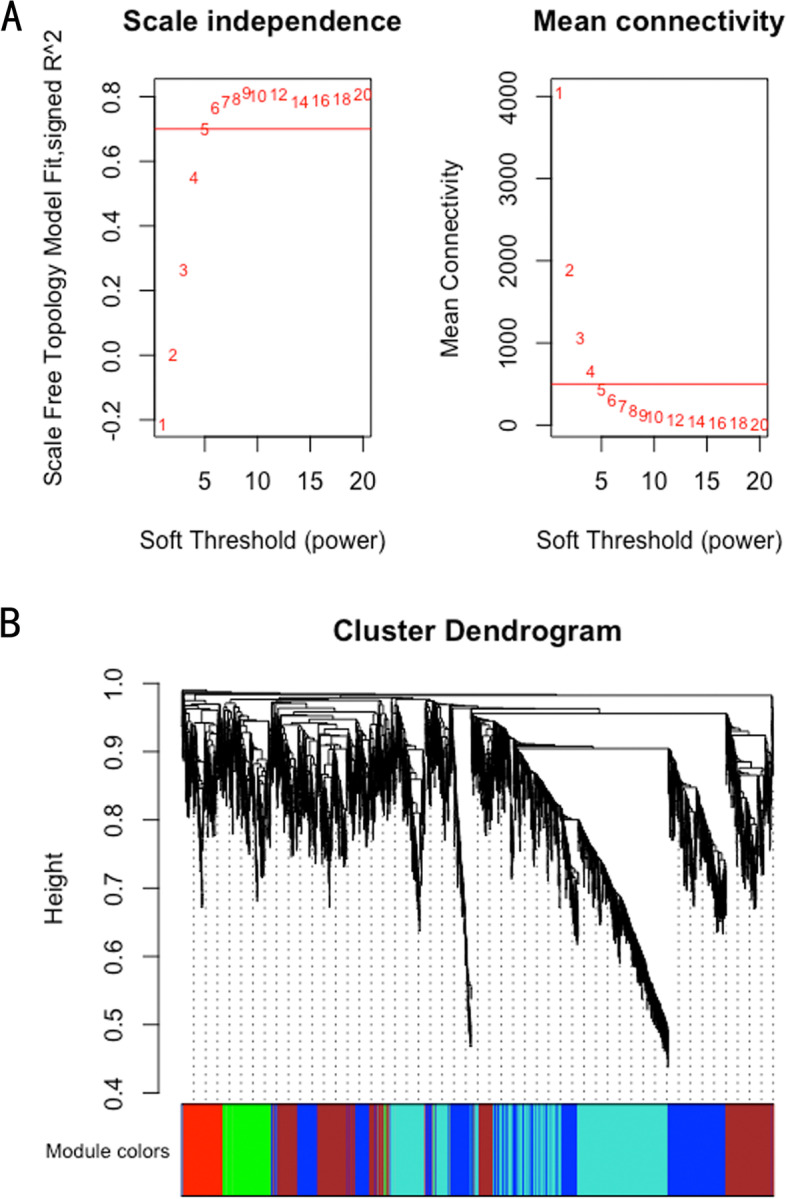
Weighted gene coexpression network analysis. **A** Scale independence and mean connectivity analysis for various soft-thresholding powers. Left panel: The *x*-axis indicates the power value, and the *y*-axis indicates the scale-independent value. Red line: The scale independent value was 0.7 when the power value was set to 6. Right panel: The *x*-axis indicates the power value, and the *y*-axis indicates the mean connectivity. **B** Coexpression modules built by hierarchical clustering and dynamic branch cutting. The dynamic tree cutting algorithm clustered similarly expressed genes into discrete branches to define modules with a minimum module size of 30 genes and a cut-height threshold of 0.25. The modules were assigned to different colors for visualization. A total of 7 modules were identified

**Fig. 5:**
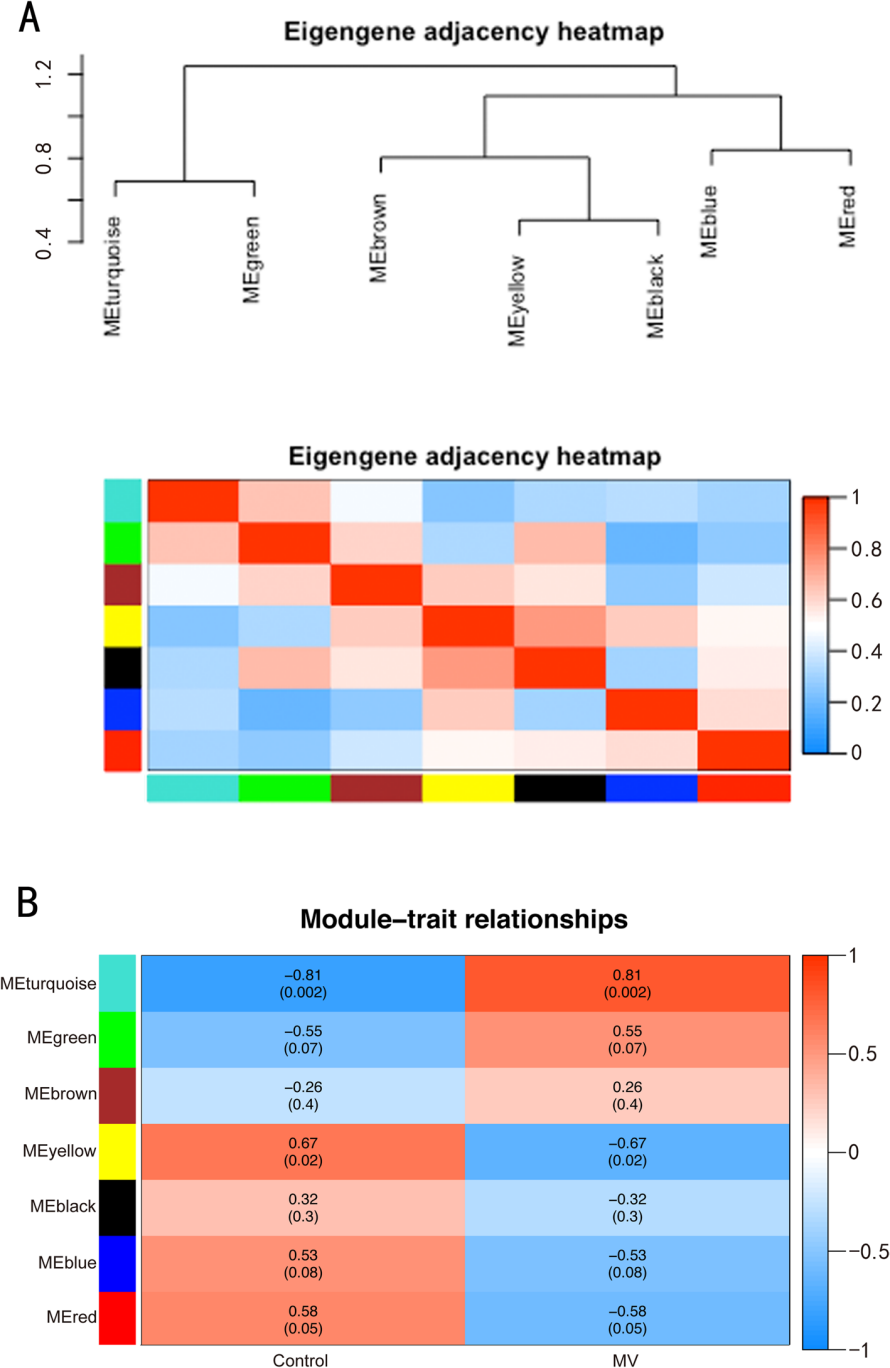
Module–trait correlations. **A** Eigengene adjacency heatmap. Eigengene adjacency heatmaps are depicted to indicate the connectivity among the coexpression modules. **B** Correlation heatmap of gene modules and group features. The turquoise and yellow modules were markedly correlated with the status of accepting mechanical ventilation (MV) and the control condition, respectively. Each column corresponds to a group feature, while each row corresponds to a module eigengene. The Pearson correlation coefficients between group features and module eigengenes and the *P* values are presented in each cell

**Fig. 6:**
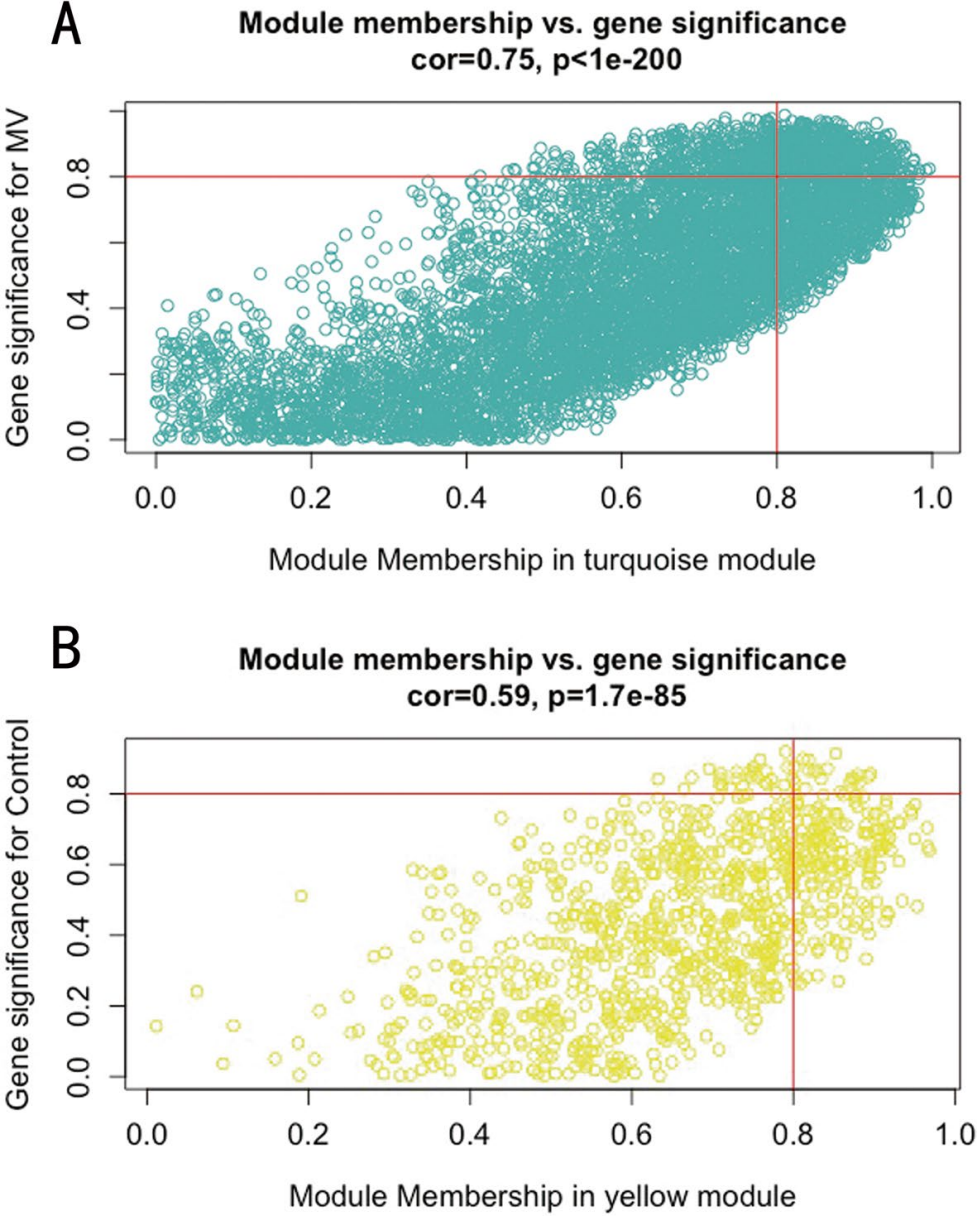
Hub genes defined by module membership (MM) and gene significance (GS). In WGCNA, GS represents the correlation between a gene and a trait. The MM represents the correlation between an individual gene and the module eigengene. GS is plotted on the *y*-axis, MM is plotted on the *x*-axis, and each point represents an individual gene within each module. **A** Forty-five hub genes were identified according to a GS >0.8 and an MM >0.8 within the turquoise module. **B** Fifteen hub genes were identified according to a GS >0.8 and an MM >0.8 within the yellow module

**Fig. 7:**
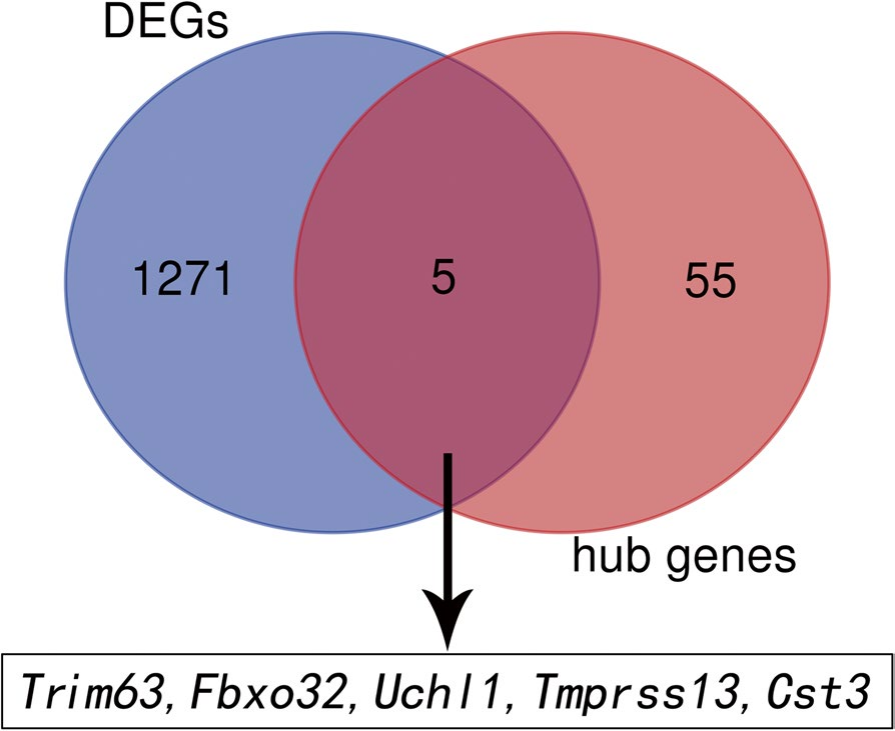
Venn diagram utilized to screen for the co-differentially expressed hub genes. The Venn diagram shows the five overlapping genes (*Trim63*, *Fbxo32*, *Uchl1*, *Tmprss13*, and *Cst3*) present among both the differentially expressed genes and the hub genes from the turquoise and yellow modules

**Fig. 8:**
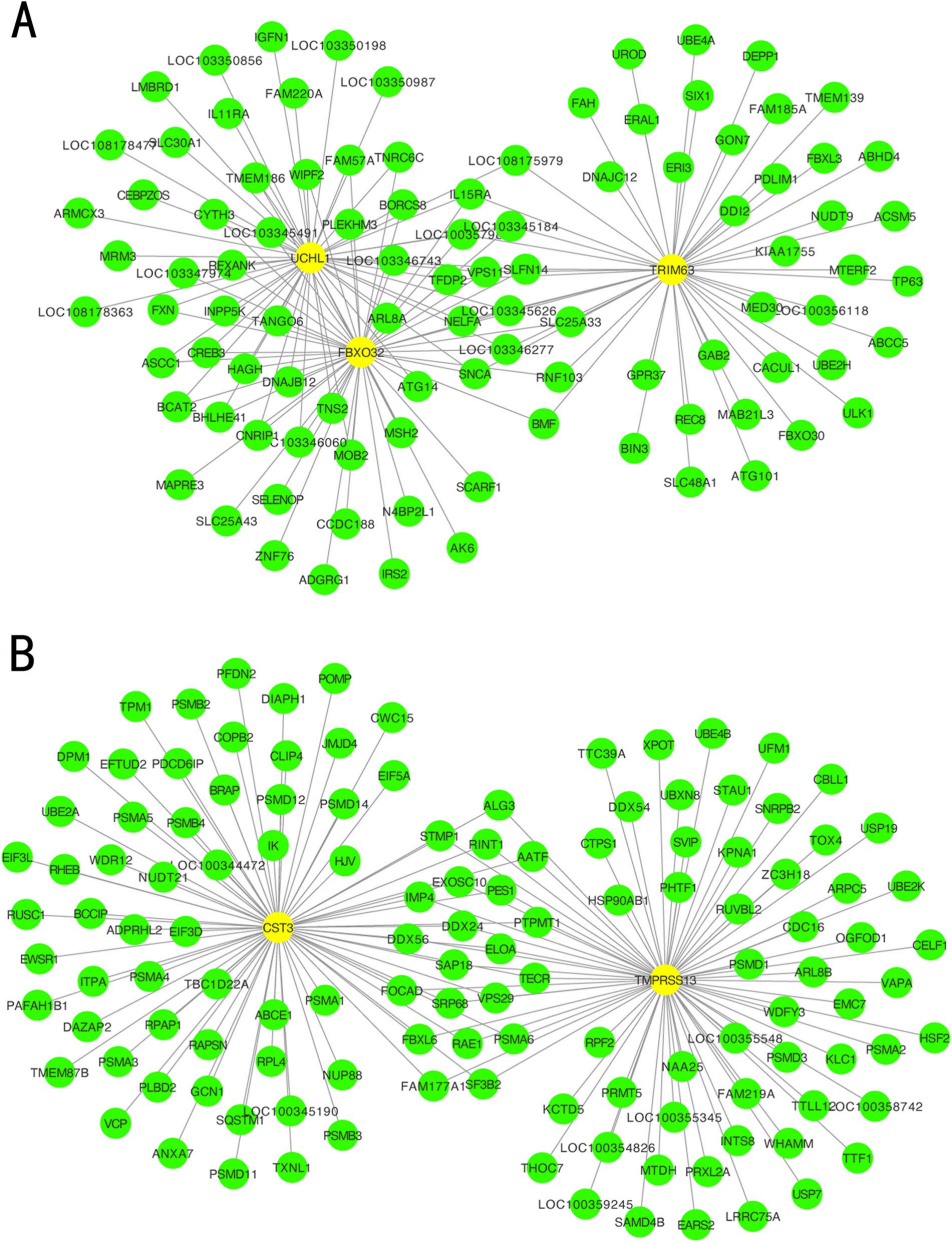
Coexpression networks of the five co-differentially expressed hub genes. A coexpression network was constructed and visualized with the five hub co-differentially expressed hub genes and their target genes in Cytoscape 3.8.2 (www.cytoscape.org). Each node represents a gene. Each edge (line) represents an interaction between two genes. The yellow nodes represent the co-differentially expressed hub genes identified by DEG and WGCNA. **A** Network with *Trim63*, *Fbxo32*, and *Uchl1* as core genes, which are closely related to the ubiquitin–proteasome system (UPS) balance. **B** The other network with *Tmprss13* and *Cst3* as core genes, showing that there is a certain degree of crosstalk between apoptosis and autophagy

WGCNA was performed for the diaphragm expression profile to screen for hub genes within the core modules involved in the progression of VIDD. Dynamic branch cutting and hierarchical clustering were applied to build coexpression modules (Fig. 4), and the relationships among seven such modules, along with their group features, are presented in Fig. 5. Of these, the turquoise module was significantly positively correlated with the status of accepting MV (*r* = 0.81, *P =*0.002), and the yellow module was significantly positively correlated with the control condition (*r*=0.76, *P* =0.02). Five co-differentially expressed hub genes (*Uchl1*, *Tmprss13*, and *Cst3*, *Fbxo32*, *Trim63*) were identified as significant candidates that were differentially expressed between the two groups in addition to the hub genes from the turquoise and yellow modules. Two of them that were related to muscle atrophy (*Fbxo32* and *Trim63*) were excluded from the study, while the remaining three (*Uchl1*, *Tmprss13*, and *Cst3*) were selected for both qRT–PCR and western blotting. The 60 key genes with high MM and GS in the turquoise and yellow modules were selected as hub genes in our study (MM>0.8; GS>0.8) (Fig. 6). As shown in the Venn diagram, five overlapping genes between the 1276 DEGs and the 60 hub genes in the turquoise and yellow modules were defined as co-differentially expressed hub genes (Fig. 7). Finally, two coexpression networks were built with the five hub co-differentially expressed hub genes and their target genes and visualized in Cytoscape (v3.8.2) (Fig. 8). One network with *Trim63*, *Fbxo32*, and *Uchl1* as core genes was closely related to the UPS balance, and the other network with *Tmprss13* and *Cst3* as core genes showed that there is a certain degree of crosstalk between apoptosis and autophagy.

### qRT–PCR and western blotting

**Fig. 9:**
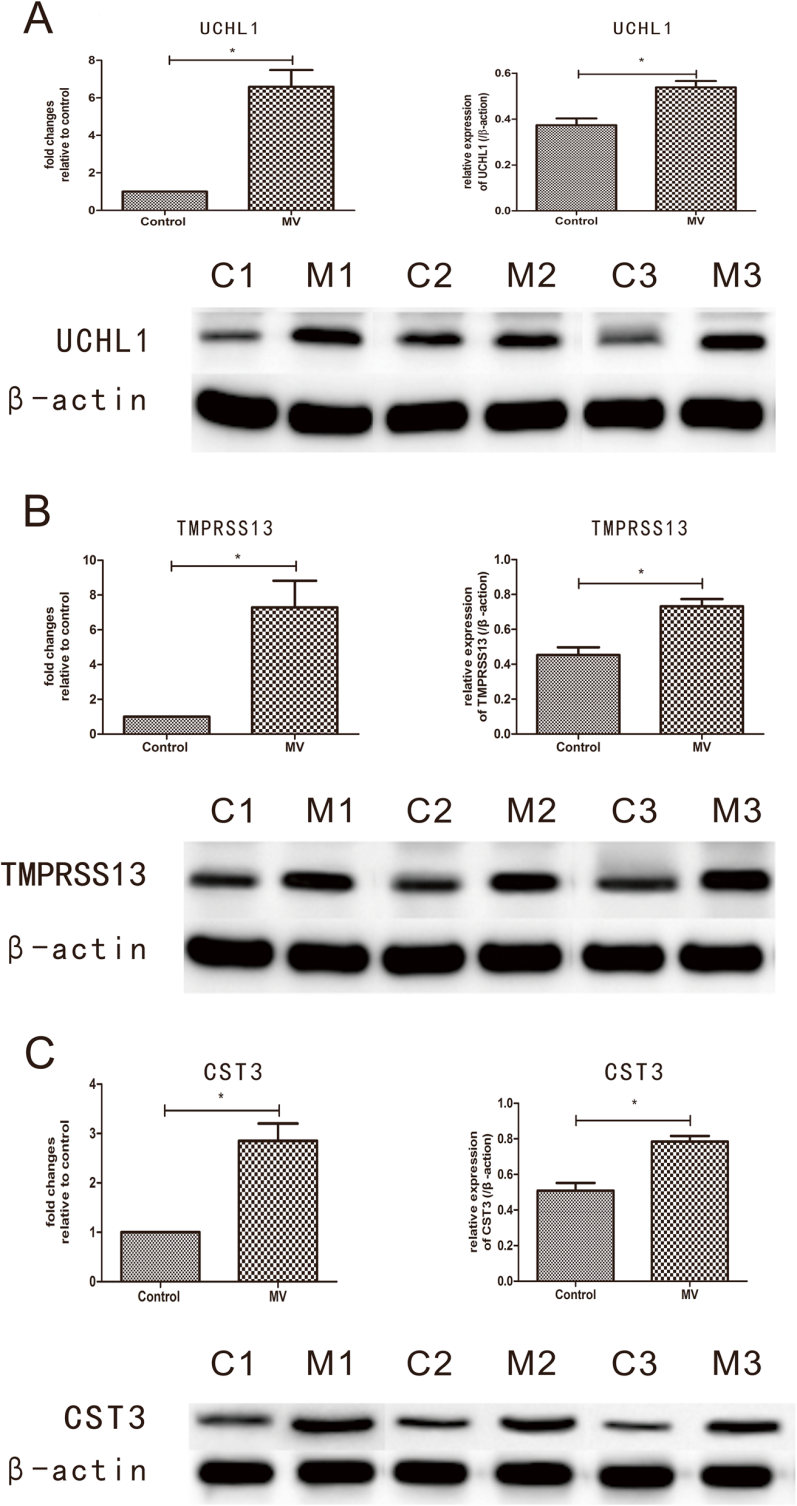
qRT–PCR and western blot analysis. **A**–**C** The top bar graph presents the mRNA/protein levels (*Uchl1*/UCHL1, *Tmprss13*/TMPRSS13, and *Cst3*/CST3) in the diaphragm for the MV and control groups. The mRNA expression levels of three genes measured by quantitative real-time PCR were normalized with respect to β-actin and are expressed as fold changes relative to the control levels. The protein expression levels were also normalized with respect to β-actin. The bottom panel presents the western blotting results for UCHL1, TMPRSS13, CST3, and β-ACTIN protein levels in the diaphragm for the MV and control groups. The data are presented as the mean ± SD (*n*=6); “*” indicates *P* < 0.05 compared with the control group

The expression levels of the selected genes/proteins, namely, *Uchl1/*UCHL1, *Tmprss13*/TMPRSS13, and *Cst3/*CST3, were validated by qRT–PCR and western blotting. The qRT–PCR and western blotting results confirmed the sequencing results. The three genes were significantly upregulated in the MV group compared with the control group (*P* < 0.01). In addition, the levels of the corresponding proteins were found to increase significantly (*P* < 0.05) in the MV group of rabbits after one day of MV (Fig. 9).

## Discussion

This study is the first to systematically investigate the endogenous protective mechanism against VIDD by identifying co-differentially expressed hub genes via WGCNA. After an MV model was successfully established in rabbits, high-throughput RNA-seq established that 1276 genes were differentially expressed after MV; among these, 760 were downregulated, while 516 were upregulated. The use of WGCNA to construct coexpression networks further helped to identify core modules and hub genes. The turquoise module was found to have the greatest correlation with the status of accepting MV, and the yellow module displayed a significant positive correlation with the control condition. Five candidate co-differentially expressed hub genes (*Uchl1*, *Tmprss13*, *Cst3*, *Fbxo32*, and *Trim63*) were ultimately identified by overlapping the DEGs and the hub genes within the core modules. Of these, two atrophy-inducing genes (*Trim63 and Fbxo32*) were excluded from the study. The remaining three genes (*Uchl1*, *Tmprss13*, and *Cst3*) were validated by qRT–PCR as well as western blotting to support the results from RNA-seq. Overall, the identification of these genes may help elucidate the physiological and pathological processes underlying VIDD.

Although traditional DEG analysis has provided a wealth of helpful information, WGCNA is arguably the only approach to uncover modules of coexpressed genes linked to clinical features. In coexpression networks, a group of genes with the same expression pattern is clustered in the same module, and the associations between modules and clinical features have been established [29]. In this study, five co-differentially expressed hub genes (*Trim63*, *Fbxo32*, *Uchl1*, *Cst3*, and *Tmprss13*) were discovered based on RNA-seq and WGCNA. During MV, a critical proteolytic system in diaphragm fibers is the UPS [12, 13], whose main function is to break down polyubiquitinated proteins. Of the five co-differentially expressed hub genes identified, *Trim63 and Fbxo32*, which encode two muscle-specific E3 ubiquitin ligases in the UPS system, have been found to promote the degradation of the molecular motor myosin in rat models of VIDD due to increased expression levels. Although the identification of these two genes supported earlier findings of their involvement in VIDD, they were excluded from further analysis [30]. Instead, the subsequent analyses focused on the remaining three genes and proteins (*Uchl1*/UCHL1, *Cst3*/CST3, and *Tmprss13*/TMPRSS13), which were not only increasingly expressed in the MV group but were also likely to reduce MV-associated physiological stress by exerting antiproteasomal, antiapoptotic, and antiautophagic effects in the diaphragm.

Ubiquitin C-terminal hydrolase 1 (UCHL1), first identified as an abundant protein in the brain, cleaves ubiquitin from small substrates. In addition, it has a crucial role in restoring ubiquitin homeostasis [31]. This protein also affects the development and function of the skeletal muscles. For instance, after denervation damage, UCHL1 levels tend to rise in response to spinal muscular atrophy in an attempt to restore ubiquitin homeostasis by preserving ubiquitin levels and avoiding uncontrolled degradation [32]. The increased UCHL1 expression in this study suggests that the protein could be important for maintaining the levels of free ubiquitin, thereby exerting a protective effect against VIDD.

Cystatin C (CST3), which is present in almost all organs of the body and biological fluids, is a highly abundant cysteine protease inhibitor. The enhanced expression of its gene could be a protective factor against the progression of VIDD. This seems reasonable considering the evolutionarily conserved process of autophagy in which cellular components are transported to lysosomes, where they are broken down and recycled [33]. Indeed, in muscle fibers, MV can induce rapid removal of cytosolic proteins and healthy organelles, thereby leading to diaphragm atrophy [15]. However, this process can be regulated by *Cst3*, a conserved gene in both humans and mice that encodes a cysteine protease inhibitor that is essential for preserving spermatogonial stem cells (SSCs). In fact, in mouse SSCs, knocking down this gene enhances autophagic activity, indicating that the corresponding protein could negatively regulate autophagy in SSCs [34]. An extrapolation of this function would suggest that increased CST3 expression, such as that observed in this study, could suppress autophagy, which is known to contribute to VIDD. Furthermore, upregulation of autophagy can induce selective degradation of the endogenous antioxidant catalase, thereby increasing ROS levels. In this case, autophagy suppression by CST3 may decrease the generation of ROS and avoid a positive-feedback loop in which autophagy, driven by oxidative stress, can lead to more ROS accumulation and autophagy.

The type II transmembrane serine protease (TTSP) subfamily includes twenty structurally distinct multidomain serine proteases and represents a relatively new classification for serine proteases that are anchored to membranes [35]. These proteins, which are expressed on the surfaces of epithelial cells of most organs, are involved in tissue homeostasis as well as the development of the epithelium. The hepsin/TMPRSS subfamily is a subclassification of the TTSP family and includes transmembrane protease serine 13 (TMPRSS13) [36], a protein whose ability to promote breast and colorectal cancer (CRC) progression in vivo due to its antiapoptotic properties has been recently reported. In this context, one study has shown that genetic ablation of *Tmprss13* can enhance apoptosis in tumor cells in mice [37]. In contrast, transgenic overexpression of *Tmprss13* results in significantly reduced cleavage of caspase-3, which subsequently causes CRC cells to develop antiapoptotic properties [38]. Based on the above, the relatively high expression of TMPRSS13 observed in this study could hint at the existence of an antiapoptotic mechanism that protects against VIDD by blocking caspase-3.

When multicellular organisms encounter cellular stress during their development, a compensatory proliferation mechanism might contribute to the recovery of tissues in order to ensure the survival of the organisms [39]. In this context, it is speculated that the high levels of UCHL1, TMPRSS13, and CST3 could be involved in the repair of diaphragmatic fibers. Testing this speculation will be helpful for understanding the progression of VIDD. UCHL1 may inhibit myoblast differentiation while promoting its proliferation, thereby contributing to muscle regeneration and repair [40]. Similarly, in the case of TMPRSS13, it has been recently reported that this protein can regenerate various tissues after injuries by converting the single-chain pro-hepatocyte growth factor (HGF) into biologically active HGF, which functions as a motogen, mitogen, and morphogen in various cells [36, 41]. Finally, in recent in vitro and in vivo studies, CST3 has been shown to exert protective effects through cell proliferation [42]. In particular, CST3 enables glomerular rat mesangial cells to proliferate in an autocrine manner [43]. Knockdown of the encoding gene in mice reduces the basal level of neurogenesis in the subgranular zone of the dentate gyrus, thus highlighting the role of this molecule in neurogenesis [44]. Despite the antiapoptotic, antiproteasomal, and antiautophagic effects speculated for the identified genes, it is likely that they are unable to completely counteract the apoptotic, proteasomal, and autophagic processes that impact diaphragm fibers during MV, thereby eventually leading to VIDD. Hence, additional research aimed at identifying means of strengthening these protective roles to restore balance could be as important as research targeting specific molecular pathways for preventing VIDD.

### Limitations

This study was not without limitations. First, although the three co-differentially expressed hub genes identified based on bioinformatics analyses (*Uchl1*, *Tmprss13*, and *Cst3*) were validated, the speculation that these genes could exert antiapoptotic, antiproteasomal, and antiautophagic effects in VIDD was based on previous results. Therefore, additional studies are required to determine the actual functions of these genes. Second, the relatively small number of animals used per group could have limited the results. Future studies with larger numbers of animals will provide more accurate and generalizable results. Third, the relationship between the repair mechanism and the compensatory response of diaphragm fibers through the actions of the three co-differentially expressed hub genes in VIDD has yet to be investigated.

## Conclusion

A combination of differential gene expression analysis and WGCNA revealed that UCHL1, TMPRSS13, and CST3 are three endogenous secreted proteins that play an important role in protecting the diaphragm against VIDD. The findings of this study further suggest that investigating the functions of the identified genes (*Uchl1*, *Tmprss13*, and *Cst3*) and their involvement in the regeneration mechanism of diaphragms in VIDD will be essential.

## Data Availability

All data generated or analyzed during this study are included in this published article.

## Abbreviations

Bim: Bcl2-interacting mediator
BSA: Bovine serum albumin
CST3: Cystatin C
CRC: Colorectal cancer
DEGs: Differentially expressed genes
GS: Gene significance
HE: Hematoxylin–eosin
HR: Heart rate
IGF: Insulin-like growth factor
MM: Module membership
MV: Mechanical ventilation
mTOR: Mammalian target of rapamycin
MSPL: Mosaic serine protease large form
ME: Module eigengene
PVDF: Polyvinylidene difluoride
PaO_2_: Arterial partial pressure of oxygen
PaCO_2_: Arterial partial pressure of carbon dioxide
qRT–PCR: Quantitative real-time polymerase chain reaction
ROS: Reactive oxygen species
RNA-seq: RNA sequencing
SR: Sarcoplasmic reticulum
SIRT1: Sirtuin1
SBP: Systolic blood pressure
SSC: Spermatogonial stem cells
TTSP: Type II transmembrane serine protease
TMPRSS13: Transmembrane protease serine 13
UPS: Ubiquitin–proteasome system
UCHL1: Ubiquitin C-terminal hydrolase 1
VIDD: Ventilator-induced diaphragm dysfunction
WGCNA: Weighted gene coexpression network analysis
WB: Western blot
